# High Concordance of E-Nose-Derived Breathprints in a Healthy Population: A Cross-Sectional Observational Study

**DOI:** 10.3390/s25082610

**Published:** 2025-04-20

**Authors:** Silvano Dragonieri, Vitaliano Nicola Quaranta, Andrea Portacci, Teresa Ranieri, Giovanna Elisiana Carpagnano

**Affiliations:** Respiratory Diseases, University of Bari “Aldo Moro”, 70121 Bari, Italy; vitalianonicola.quaranta@policlinico.ba.it (V.N.Q.); andrea.portacci@uniba.it (A.P.); teresa.ranieri@uniba.it (T.R.); elisiana.carpagnano@uniba.it (G.E.C.)

**Keywords:** exhaled breath analysis, electronic nose, Cyranose 320, VOCs, breathprints, standardization, diagnostics

## Abstract

**Highlights:**

**What are the main findings?**

- E-nose breathprints in healthy subjects exhibited high concordance, with 138 out of 139 individuals clustering closely together, demonstrating low variability.
- Principal Component Analysis (PCA) explained 97.15% of the variance, reinforcing the stability of e-nose-based breath signatures under controlled conditions.

**What is the implication of the main finding?**

- This study supports the feasibility of e-nose standardization, as breathprints from healthy individuals are reproducible and show minimal variability.
- Findings pave the way for clinical applications, suggesting that e-nose technology could reliably differentiate between normal and pathological breath signatures.

**Abstract:**

Exhaled breath analysis using electronic noses (e-noses) is a promising non-invasive diagnostic tool. However, a lack of standardized protocols limits clinical implementation. This study evaluates the consistency of breathprints in healthy subjects using the Cyranose 320 e-nose to support standardization efforts. Breath samples from 139 healthy non-smoking subjects (age range 18–65 years) were collected using a standardized protocol. Participants exhaled into a Tedlar bag for immediate analysis with the Cyranose 320. Principal Component Analysis (PCA) was used to reduce data dimensionality, and K-means clustering grouped subjects based on breathprints. PCA identified four principal components explaining 97.15% of variance. K-means clustering revealed two clusters: 1 outlier and 138 subjects with highly similar breathprints. The median distance from the cluster center was 0.21 (IQR: 0.18–0.24), indicating low variability. Box plots confirmed breathprint consistency across subjects. The high consistency of breathprints in healthy subjects supports the feasibility of standardizing e-nose protocols. These findings highlight the potential of e-noses for clinical diagnostics, warranting further research in diverse populations and disease cohorts.

## 1. Introduction

Exhaled breath analysis is an emerging tool in the field of personalized medicine, offering a non-invasive means of detecting and monitoring diseases such as asthma, chronic obstructive pulmonary disease (COPD), and various types of cancer [1]. The detection of volatile organic compounds (VOCs) in exhaled breath provides valuable insights into metabolic processes, inflammation, and oxidative stress within the body [2].

Techniques such as gas chromatography–mass spectrometry (GC-MS), proton transfer reaction–mass spectrometry (PTR-MS), and selected ion flow tube–mass spectrometry (SIFT-MS) enable precise VOC detection [3]. While GC-MS requires specialized expertise and prolonged analysis time, PTR-MS provides real-time VOC profiling and is portable, making it feasible for point-of-care applications [4]. However, these techniques still lack widespread clinical adoption due to cost and infrastructure constraints.

To address these limitations, electronic noses (e-noses) have been developed as portable and user-friendly devices capable of detecting VOC patterns in exhaled breath. E-noses consist of sensor arrays that respond to various VOCs by generating unique electronic signals, which are then processed using machine learning algorithms to identify disease-specific patterns [4]. By bridging the gap between exhaled VOC analysis and practical clinical application, e-noses offer a promising solution for point-of-care diagnostics.

The clinical relevance of exhaled breath analysis extends beyond respiratory diseases [5]. VOC profiles can also reveal metabolic disturbances, infectious diseases, and even malignancies [6]. This highlights the potential of e-noses to serve as diagnostic tools across a wide spectrum of medical conditions, providing early detection and monitoring of disease progression.

A recent European Respiratory Society (ERS) task force document provided guidelines aimed at homogenizing the various methodological aspects of breath sampling and e-nose analysis [7]. However, the lack of standardization in breath sampling and data interpretation poses a significant barrier to the clinical adoption of e-nose technology. Factors such as environmental conditions, device calibration, and patient preparation can all impact the reliability of breath analysis [8]. Addressing these challenges is essential for the successful integration of e-nose technology into routine clinical practice.

Recent advancements in sensor technology and data analysis methods have aimed to improve the accuracy and consistency of e-nose measurements. Machine learning algorithms, for example, have been employed to enhance pattern recognition in VOC profiles, thereby improving diagnostic accuracy [4]. Despite these advancements, a consensus on standard operating procedures for breath analysis remains elusive.

Based on the above, the aim of this study was to address the issue of consistency in e-nose measurements by analyzing breathprints from a cohort of 139 healthy subjects using the Cyranose 320. We employed advanced statistical techniques to evaluate the degree of homogeneity in the breathprints. The ultimate goal is to lay the groundwork for the standardization of e-nose methods, thereby enhancing their reliability and reproducibility in clinical settings.

## 2. Materials and Methods

### 2.1. Study Population

A total of 139 healthy subjects were recruited for this study. Participants ranged in age from 18 to 65 years, with a balanced representation of genders. The median age was 42 years, with 52% identifying as female and 48% as male (Table 1). Inclusion criteria required that participants be non-smokers with no history of chronic respiratory, metabolic, or infectious diseases. Subjects with recent upper respiratory infections, chronic use of medication affecting respiratory function, or any condition that could alter breath composition were excluded from this study. Applicants were recruited from hospital staff. Each subject provided informed consent, and the study protocol was approved by the institutional ethics committee of Policlinico di Bari (protocol number 46403/15).

### 2.2. Breath Sampling Procedure

Breath samples were collected using a standardized protocol to minimize variability. To diminish the influence of potential confounding factors, such as recent dietary intake, oral hygiene products, and circadian variability in breath composition, all participants were instructed to abstain from eating, drinking (except water), smoking, and using oral care products for at least three hours before breath sampling. Participants were also evaluated in the morning to reduce inter-subject variability related to daily activities. Moreover, the breath collection process was performed under controlled environmental conditions, with temperature and humidity maintained within a defined range to reduce external influences on VOC detection. The procedure for collecting exhaled breath involved having participants wear a nose clip and breathe naturally at a resting rate for a duration of five minutes through a two-way valve (Hans Rudolph 2700, Hans Rudolph, Shawnee, KS, USA) that was attached to an inspiratory filter to reduce environmental volatile organic compounds contamination (A2, North Safety, Middelburg, The Netherlands). Following this, each individual performed a full exhalation to vital capacity into a Tedlar bag, which was then immediately analyzed using the e-nose.

### 2.3. Electronic Nose

For the current study we used a Cyranose 320 (Sensigent, Baldwin Park, CA, USA), a portable e-nose device designed to detect and classify VOCs in various samples, including exhaled breath. The Cyranose 320 utilizes a proprietary sensor array technology composed of 32 nano-composite organic polymer sensors. Each of these sensors responds to specific VOCs by undergoing a change in electrical resistance, thereby generating a unique pattern or “breathprint” that can be analyzed for diagnostic purposes. According to the instruction manual, the device was set to operate with a sampling period of 60 s and a purge phase lasting 200 s, and it was maintained at a temperature of 42 °C.

### 2.4. Standardization Measures

To ensure methodological rigor and minimize variability, we adopted a comprehensive standardization protocol throughout this study. Breath sampling was performed under controlled environmental conditions, with temperature and humidity maintained within a defined range. All participants were instructed to refrain from eating, drinking (except water), smoking, or using oral hygiene products for at least three hours prior to sample collection. Breath was collected using a nose clip and a two-way non-rebreathing valve connected to a VOC filter, followed by exhalation into a Tedlar bag for immediate analysis, thereby eliminating the need for sample storage. The Cyranose 320 device was preconditioned and calibrated according to the manufacturer’s specifications before each use. This protocol was designed to reduce the impact of external confounders such as ambient VOCs, hydration status, and oral contamination, thereby ensuring consistency and reproducibility of breathprints across the study population.

### 2.5. Data Analysis

The raw sensor data were analyzed using SPSS software (version 26.0) to identify patterns in the VOC profiles. Principal Component Analysis (PCA) was employed to reduce the dimensionality of the dataset, transforming the data into four principal components (PCs) that accounted for 97.15% of the total variance. This dimensionality reduction was essential for simplifying complex datasets and minimizing noise, making it easier to identify meaningful patterns. K-means clustering was then applied to group subjects based on their PCA scores. The decision to use two clusters was informed by previous studies suggesting limited variability in healthy populations. K-means clustering is a widely used unsupervised machine learning algorithm that partitions data into clusters by minimizing the distance between each data point and the centroid of its assigned cluster. This approach enabled the identification of homogeneous breathprint groups within the dataset. The distances from the cluster centers were calculated to assess the proximity of each subject to their respective cluster. This analysis provided insight into the consistency of the breathprints, with smaller distances indicating greater similarity within clusters. In this study, sample size estimation was not based on traditional power calculations due to the exploratory nature of the machine learning methods used. Instead, the adequacy of the sample size was confirmed through cross-validation, dimensionality reduction via Principal Component Analysis (PCA), and clustering stability analysis. The PCA accounted for 97.15% of the variance in the dataset, and the K-means clustering results showed a cohesive grouping of patients, with minimal variability within clusters.

To determine the optimal number of clusters for K-means analysis, we employed two complementary techniques: the Elbow Method and Silhouette Analysis.

The Elbow Method was used to evaluate the relationship between the number of clusters (k) and the Within-Cluster Sum of Squares (WCSS), which quantifies the compactness of each cluster. For each value of k (ranging from 2 to 6), we computed the total WCSS, representing the sum of squared distances between each data point and its respective cluster centroid. These values were plotted against increasing numbers of clusters. The “elbow point” of the curve—where the rate of WCSS reduction markedly slows—was interpreted as the optimal number of clusters, representing a trade-off between model complexity and explanatory power.

Silhouette Analysis was applied to assess the quality of clustering by calculating the silhouette coefficient for each data point. This metric measures how similar a subject is to its own cluster (cohesion) compared to the nearest alternative cluster (separation). Silhouette values range from −1 (poor fit) to +1 (ideal fit), with higher average scores indicating better-defined clusters. We calculated the average silhouette coefficient across all subjects for k values between 2 and 6. The highest mean silhouette score was observed at k = 2, suggesting that the data form one cohesive group with minimal variability and that any additional clusters likely represent true outliers.

The combination of these two methods allowed us to robustly identify the appropriate number of clusters and to confirm the internal homogeneity of the dataset.

To validate the stability and generalizability of the clustering, the dataset was randomly divided into a training set (50%) and a test set (50%). K-means clustering (k = 3) was performed on the training set, and the resulting cluster centroids were applied to the test set. The clustering quality in the test set was assessed using the silhouette score and analysis of cluster distribution.

A *p*-value < 0.05 was considered as significant.

## 3. Results

The PCA results indicated four principal components that collectively explained 97.15% of the variance in the dataset. Component 1 captured 80.58% of the variance, Component 2 accounted for 8.58%, Component 3 explained 6.77% of the variance, while Component 4 contributed 1.22% (Table 2).

These components were essential in simplifying the dataset by reducing noise and retaining the most informative variables. By transforming the complex sensor data into principal components, we could better visualize and interpret the patterns within the breathprints.

K-means clustering was performed using the four principal components to group subjects based on their breathprints. Two clusters were then identified.

Cluster 1 contained one subject with unique characteristics that deviated significantly from the rest of the population. Cluster 2 comprised 138 subjects, indicating a high degree of similarity in their VOC profiles (Table 3 and Table 4).

**Figure 1:**
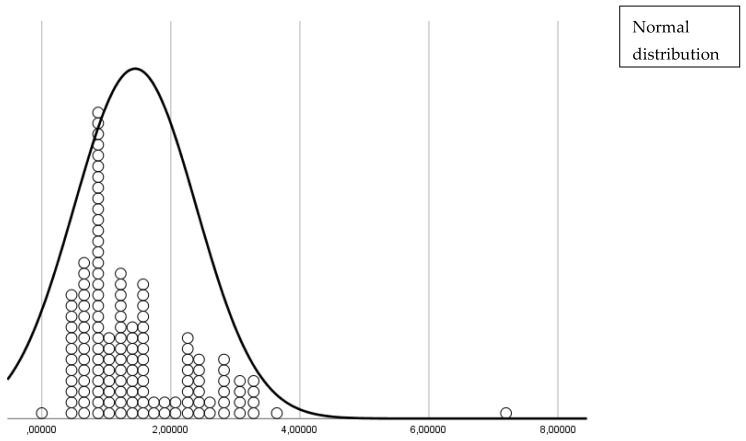
Distances from the center of each cluster for all patients, showing the relative proximity of each patient to the center of their assigned cluster.

**Figure 2:**
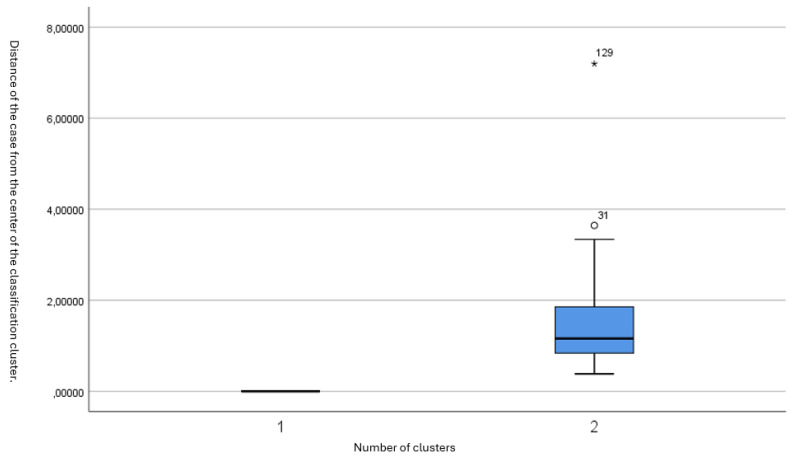
Box plot of the distances from the cluster center, demonstrating that the majority of patients have small distances from the midpoint.

The distribution of subjects within each cluster is depicted in Figure 1 and Figure 2. Figure 1 shows the distances of each subject from the cluster center, highlighting that the majority were tightly grouped around the center of Cluster 2. Figure 2 presents a box plot illustrating the low variability within Cluster 2 and the distinct separation of the outlier in Cluster 1.

**Figure 3:**
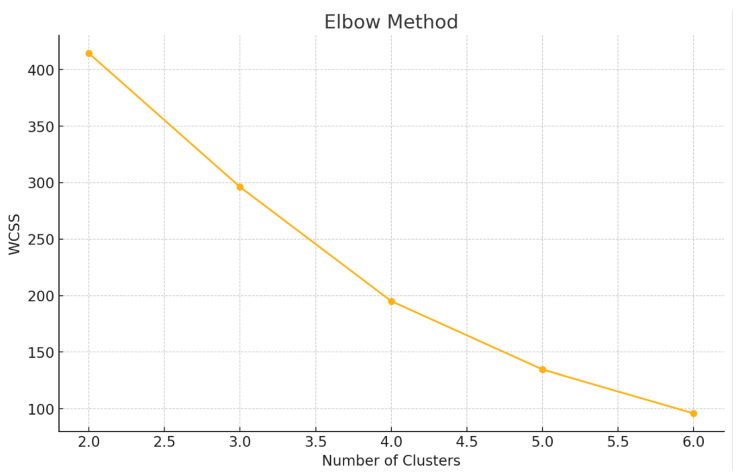
Elbow plot to determine the optimal number of clusters.

To determine the optimal number of clusters, both the Elbow Method and Silhouette Analysis were applied to the four principal components derived from PCA. The Elbow plot (Figure 3) showed a distinct change in slope between k = 2 and k = 3, indicating a point of diminishing returns beyond two or three clusters. This “elbow’ suggests that adding more clusters beyond this point does not significantly reduce the Within-Cluster Sum of Squares (WCSS), supporting a more parsimonious model.

**Figure 4:**
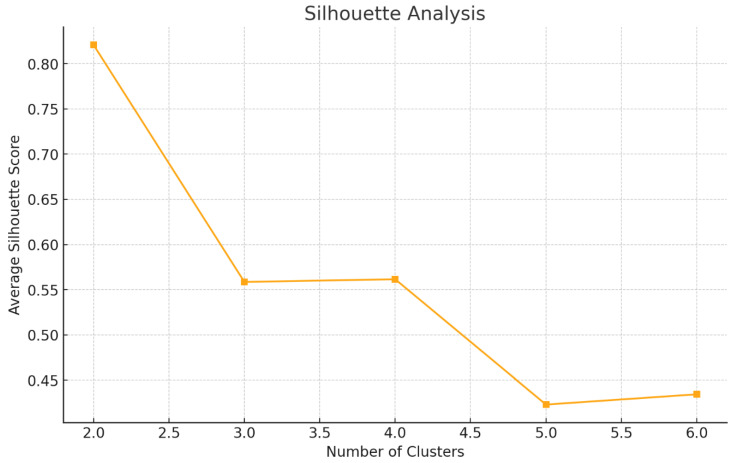
Silhouette Analysis to determine the optimal number of clusters.

Silhouette Analysis (Figure 4) revealed the highest average silhouette score at k = 2 (0.82), with a modest decline to 0.56 at k = 3. This indicates that the best cluster cohesion and separation occur with two clusters, consistent with a population characterized by highly homogeneous breathprints. When k = 3 was tested, the vast majority of subjects (137 of 139) remained grouped in a single dominant cluster, with the remaining 2 individuals forming distinct, small clusters. This pattern further confirms the overall low inter-individual variability in VOC profiles among healthy subjects, with only minimal deviation attributable to potential biological or technical outliers.

In the internal validation procedure using a train/test split, K-means clustering was trained on the training set and applied to classify the test set. The average silhouette score in the test set was 0.52, confirming good cohesion and separation. Cluster distribution in the test set remained stable (Cluster 1: 51 subjects; Cluster 2: 11; Cluster 3: 8), confirming that the clustering structure was generalizable and not driven by overfitting.

## 4. Discussion

The primary finding of this study is the high degree of homogeneity in breathprints among normal subjects when measured using the Cyranose 320. The clustering analysis indicated that nearly all subjects were classified within a single cluster, suggesting consistent VOC profiles across the cohort. This consistency is essential for the potential standardization of e-nose methodologies and suggests that healthy individuals share similar VOC patterns.

To the best of our knowledge, this is the first study to comprehensively assess the consistency of exhaled breathprints in a quite large cohort of healthy subjects using e-nose technology, with a specific focus on cluster analysis to explore VOC profile homogeneity.

Although the primary clinical goal of e-nose technology is the early detection of disease, establishing a robust baseline in healthy individuals is a fundamental prerequisite. The present study aimed to characterize the reproducibility and internal consistency of breathprints under standardized conditions, thus laying the groundwork for future studies involving patients with respiratory or systemic diseases.

To ensure the robustness of our statistical approach, we employed Principal Component Analysis to reduce dimensionality and applied K-means clustering to categorize breathprints. The use of four principal components explained 97.15% of the variance in the dataset, indicating that the chosen components captured most of the relevant information.

Our group has previously conducted studies investigating the reproducibility of exhaled VOC profiles and the influence of factors such as sampling conditions, demographic variables, and respiratory function on breath analysis using electronic nose technology [9,10,11]. These studies demonstrated that exhaled breathprints remain stable over time and are not significantly affected by variables like age, gender, or repeated measurements [9,10,11]. Following these previous investigations, the present study confirms that breathprints among healthy subjects exhibit a high degree of consistency, further demonstrating the feasibility of standardizing e-nose methodologies for clinical applications.

Our findings align with previous studies using GC-MS in healthy subjects, which demonstrated some degree of consistency in the identification of key volatile organic compounds, VOCs, across individuals [12,13]. Specifically, Yamanaka et al. showed that certain VOCs, such as acetic acid, dimethoxymethane, n-hexane, and benzoic acid methyl ester, were consistently detected across all subjects and visits, indicating stability in these compounds over time [12]. Additionally, Jia et al. highlighted that while demographic factors influenced VOC levels, certain compounds like acetone and isoprene displayed relatively consistent patterns across the population [13]. These findings collectively suggest that specific VOCs can serve as stable biomarkers in exhaled breath, despite some variability influenced by individual and environmental factors.

We acknowledge that environmental VOCs can influence exhaled breath composition and may act as confounding factors, even under controlled sampling conditions. Although we attempted to mitigate this risk by using a standardized protocol—including the use of an A2 VOC filter (North Safety, NL) and sampling in a fixed, well-ventilated clinical environment—we recognize that real-time chemical monitoring of ambient VOCs was not performed. Future studies should consider assessing the variability of breathprints under different environmental conditions (e.g., urban vs. rural settings, indoors vs. outdoors) and at varying times of day to quantify the potential influence of ambient air composition. Moreover, direct characterization of environmental VOCs using techniques such as GC-MS would allow for a deeper understanding of their impact on breath analysis and help refine standard operating procedures for breath-based diagnostics.

One potential limitation of our study might be that clustering algorithms such as K-means are sensitive to the initial choice of centroids. To mitigate this concern, we repeated the K-means analysis with different initializations to ensure that our clusters were stable and reproducible. Furthermore, we tested alternative clustering methods, including hierarchical clustering, and found consistent results, which strengthens the reliability of our findings. While the present study employed unsupervised techniques—namely Principal Component Analysis (PCA) and K-means clustering—to explore the intrinsic structure and variability of breathprints in a healthy population, we recognize that various supervised and unsupervised machine learning (ML) methods have shown promise in breathomics. Techniques such as Support Vector Machines (SVMs), Random Forests, and neural networks have been successfully applied in disease classification tasks involving exhaled VOC profiles [14]. However, these approaches require labeled datasets and are most appropriate when comparing diseased and control populations. Our focus in this study was to establish a baseline of breathprint reproducibility under standardized conditions, which is a prerequisite before applying supervised classification algorithms in clinical cohorts. Future studies from our group will aim to incorporate and compare different ML models to evaluate their diagnostic accuracy across diverse populations and pathological conditions.

Another potential criticism is the sample size of 139 subjects. While this is a reasonable number for an exploratory study, larger datasets would provide more power to detect subtle differences in breathprints. Future studies should aim to increase the sample size and include diverse populations to enhance the generalizability of the findings.

A further limitation of this study is the focus on a healthy population. Future research should include patients with various respiratory and metabolic diseases to determine whether the observed homogeneity holds across different clinical conditions. Additionally, the use of a single e-nose device (the Cyranose 320) limits the applicability of our findings. Comparative studies involving multiple devices could yield significant understanding of inter-device variability and the need for device-specific calibration protocols.

One notable limitation of this study is the lack of gas chromatography–mass spectrometry (GC-MS) analysis to identify the specific VOCs contributing to the breathprints detected by the Cyranose 320. While the e-nose technology provides a rapid and non-invasive method for detecting VOC patterns, it does not offer the chemical specificity of GC-MS, which is considered the gold standard for VOC identification. We fully acknowledge that complementary analytical techniques such as GC-MS are essential for compound-level validation of VOC profiles. Although beyond the scope of this study, future investigations combining e-nose analysis with GC-MS will be critical to enhance chemical specificity and support the translational potential of breath-based diagnostics. Thus, incorporating GC-MS analysis will be essential to validate the VOC patterns and identify the specific compounds that may serve as biomarkers for various physiological or pathological conditions.

The results of this study suggest that standardized e-nose protocols could be developed for use in clinical practice. E-nose devices like the Cyranose 320 have the potential to serve as rapid, non-invasive screening tools in primary care settings. For example, breath analysis could be used to identify early signs of lung diseases, including COPD and lung cancer, thereby facilitating timely intervention [1].

Moreover, the consistency of breathprints observed in this study could enable longitudinal monitoring of patients. By establishing a baseline breathprint for an individual, clinicians could detect deviations that may indicate disease progression or response to treatment. This personalized approach could improve patient outcomes by allowing for more tailored interventions.

To build on the findings of this study, future research focused on standardization should include diverse populations, including patients with known respiratory diseases, to assess the diagnostic accuracy of the e-nose across various conditions as well as exploring the integration of machine learning algorithms to improve pattern recognition and enhance the predictive accuracy of e-nose devices. Further investigations should also be concentrated on the long-term stability of breathprints and the potential for e-nose devices to monitor disease progression or treatment response.

To conclude, the analysis of exhaled breath using the Cyranose 320 revealed a high degree of similarity among breathprints in a cohort of 139 normal subjects. These findings support the feasibility of standardizing e-nose methods, which is essential for their clinical application. Standardized protocols and further research involving diverse populations are needed to enhance the reliability and utility of e-nose technology in healthcare. Addressing current limitations will pave the way for the successful integration of breath analysis into routine clinical practice. From a cost perspective, the Cyranose 320 offers a relatively affordable solution, with per-test costs generally below EUR 10, excluding capital and maintenance. This contrasts with higher costs associated with mass-spectrometry-based approaches, which often require expensive consumables and operator expertise.

## Figures and Tables

**Table 1:** Clinical characteristics of the studied population.

Parameter	Value
Subjects (n.)	139
M/F (n.)	79/60
Age (y.)	37.1 ± 10.6
FEV1%pred.	101.3 ± 9.7
BMI (kg/m^2^)	26.1 ± 2.9
smokers (n.)	0
Comorbidities (n.)	0

**Table 2:** Principal Component Analysis on the dataset.

Component	Initial Eigenvalues	% of Total Variance	Cumulative %
1	25.786	80.580%	80.580%
2	2.746	8.582%	89.163%
3	2.166	6.770%	95.933%
4	0.390	1.217%	97.150%

**Table 3:** Cluster analysis showing the regression factor scores for the principal components of breathprints in the two identified clusters.

Cluster	1	2
REGR factor score 1 for analysis 1	0.85954	−0.00623
REGR factor score 2 for analysis 1	11.47740	−0.08317
REGR factor score 3 for analysis 1	−0.40734	0.00295
REGR factor score 4 for analysis 1	1.98722	−0.01440

**Table 4:** Cluster distribution and the number of cases within each identified cluster.

Cluster	Number of Cases
1	1000
2	138,000
Valid	139,000
Missing	0

## Data Availability

Raw data are available upon reasonable request.

## Abbreviations

The following abbreviations are used in this manuscript:

FEV1	Forced Expiratory Volume in the 1st Second
BMI	Body Mass Index
REGR	Regression
